# Risk literacy assessment of general practitioners and medical students using the Berlin Numeracy Test

**DOI:** 10.1186/s12875-020-01214-w

**Published:** 2020-07-14

**Authors:** Hendrik Friederichs, Roman Birkenstein, Jan C. Becker, Bernhard Marschall, Anne Weissenstein

**Affiliations:** 1 Study Hospital Münster, Institute for Education and Student Affairs, Medical Faculty of Münster, Malmedyweg 17-19, D-48149 Münster, Germany; 2 Department of Internal Medicine, Marien-Hospital, Erftstadt, NRW Germany

**Keywords:** Risk literacy, Mass screening, Mammography, Medical education, Predictive value

## Abstract

**Background:**

The responsibility for helping patients understand potential health benefits and risks, especially regarding screening tests, falls largely to general practitioners (GPs). The Berlin Numeracy Test (BNT) specifically measures risk literacy (i.e., the ability to understand different aspects of statistical numeracy associated with accurate interpretation of information about risks). This study explored the association between risk literacy levels and clinical experience in GPs vs. medical students. Additionally, the effect of GP risk literacy on evaluation of the predictive value of screening tests was examined.

**Methods:**

The participants were 84 GPs and 92 third-year medical students who completed the BNT (total score range 0–4 points). The GPs received an additional case scenario on mammography screening as a simple measure of performance in applying numeracy skills.

**Results:**

Despite having an average of 25.9 years of clinical experience, GPs scored no better than medical students on risk literacy (GPs: 2.33 points, 95% confidence interval [CI] 2.08–2.59; students: 2.34, 95% CI 2.07–2.61; *P* = .983). Of all GPs, 71.6% (*n* = 58) greatly overestimated the real predictive value.

**Conclusions:**

In this study, we found no difference in risk literacy between current students and current GPs. GPs lack risk literacy and consequently do not fully understand numeric estimates of probability in routine screening procedures.

## Background

As previously described in detail [1], several studies suggest that patients want to be included in discussions about their health, diagnostics, and therapy [2].This patient-centered and shared decision-making approach between patients and doctors is the basis of modern medicine [3–7]. Individual risk calculations are an effective method of risk communication, particularly in patient education for screening programs. Previous research has shown that adequate risk communication can even increase the use of screening tests [8]. However, some studies have demonstrated lownumeracy, the ability to perform basic quantitative calculations, among patients [9]. Because understanding risk requires a certain level of numeracy, patients must often depend on their providers to interpret and communicate risk information. If providers also do not understand the quantitative aspects of risk, patients will be unable to comprehend or use the information well [10]. It is therefore safe to assume that, as a rule, only physicians with adequate training will be able to assist their patients in this way.

Sufficient competence in statistical numeracy is essential to fully understand and communicate numeric estimates of probability [11]. The ability to understand statistics and the quantitative aspects of research varies tremendously among physicians and medical students [12, 13]. Additionally, because risk information is primarily quantitative, these individual differences arguably affect medical professionals’ comprehension of diagnostic tests and other risk-related communications [14]. The Berlin Numeracy Test [BNT] [15] is a multiple-choice instrument that specifically measures risk literacy; that is, the ability to understand different aspects of statistical numeracy associated with accurate interpretation of information about risks. The BNT was developed for use with educated and highly educated samples. In a previous study, we showed that the average risk literacy in medical students is slightly above average (Cohen’s d = .23) and does not increase during medical studies [1]. Little is known about whether risk literacy increases during postgraduate medical education.>

Previous research has suggested suboptimal risk literacy in clinicians. Gigerenzer and colleagues [16] studied 160 gynecologists and found that one-third were ignorant of the benefits of mammography screening. Most of the investigated gynecologists were unable to calculate the positive predictive value, so their probability of choosing the best answer was “slightly less than chance.” In a different report published as a letter, Estrada et al. [17] assessed the numeracy skills of 45 health professionals (including physicians, nurses, doctorate faculty, and medical students). Twenty percent of the participants gave incorrect answers to two or more of six absolutely basic numeracy questions. Bramwell and colleagues [18] reported that only about one-third of obstetricians were able to calculate the probability of an unborn child actually having trisomy 21, given a positive test result. These findings suggest that a lack of risk literacy may account for medical students and novice physicians repeating unnecessary diagnostics [19], and also may lead to an overestimation of the value of diagnostic findings.

To our knowledge, very limited data exist on the risk literacy of general practitioners (GPs), despite the importance of their role in health services in general and in risk communication in particular. GPs are the first line of contact for questions about whether to use offered screenings; therefore, they bear the main responsibility for helping patients understand the potential benefits and risks of screenings. For example, in Germany, mammography screening is offered to all women aged 50 to 69 years. The invitation includes a recommended appointment at one of the certified mammography centers. In a patient-centered study about understanding the risks and benefits of breast cancer screening, most participants overestimated the benefits. Patients with higher numeracy assessed their risk of death with and without mammography more accurately than did patients with lower numeracy [20]. In Germany, the mammography participation rate is about 56%, so nearly half of the invited women decline the recommended appointment [21]. Therefore, an additional issue that needs to be addressed in this context is the consequences of GPs’ risk literacy for their comprehension of and recommendations for screening tests.

### Research questions

We performed a controlled study of GPs and medical students to investigate the following questions:

1. What is the risk literacy among GPs?
2. Is there a difference in risk literacy levels between clinically experienced GPs and medical students?
3. Does the level of risk literacy influence evaluation of the predictive value of screening tests?

## Methods

### Setting and subjects

Our study was conducted in the winter semester of 2015/2016 with GPs of the Westfalen-Lippe State Chamber of Physicians, which is a registered corporation under public law and represents the interests of 42,000 physicians in this area (the fourth-largest state chamber in Germany). A postal questionnaire was our only financially viable option, but the response rates of GPs to postal surveys are often relatively low [22]. Expecting a large non-response rate, we contacted as many GPs as necessary to reach the a priori calculated sample size. The students were recruited at the medical school of the Westphalian Wilhelms University in Münster by approaching them in the context of a curricular course in physical examination. This course prepares students for their clinical clerkships, so it takes part at the very beginning of their clinical experience.

### Study design

The participants were asked to complete the BNT voluntarily and anonymously. The GPs received an additional case scenario on mammography screening to assess their performance in applying numeracy skills. One BNT item measures the same concept as the additional scenario (i.e., positive prediction in item 1), but in a non-clinical context. Students were not asked to complete the scenario because it is already used twice in the medical curriculum to explain two aspects of mass screening (i.e., positive predictive power and overdiagnosis).

We did not ask medical students and GPs about their academic background in statistics and probability theory. However, completion of some statistical coursework is required before taking the preliminary medical exam (“Physikum”), which is a prerequisite for entering the clinical phase of medical school. Therefore, we can assume that all participants had received statistical training.

 All participants had to agree verbally to participate. Additionally, they provided informed consent prior to the study by reading the background information and choosing to provide data. Ethical approval was given by the Ethics Committee of the Chamber of Physicians at Westfalen-Lippe and the Medical School of Westphalian Wilhelms University in Münster (2015–308-f-S).

### Data collection

In the BNT, participants were required to solve four short case studies; two gave probabilities in relative number format and two gave probabilities in absolute number format in the form of Bayesian inferences. To obtain the correct solution for these text problems, statistical information on the prevalence of a certain event had to be inserted into Bayes’ theorem. Participants received one point per correct solution; thus, their total score could range from 0 to 4 points. The test format has been previously validated by Cokely and colleagues [15] against the original paper-and-pencil version of the BNT, which in turn has been benchmarked against several population datasets [15]. The average score (standard deviation [SD]) in the original validation study with this test format is 1.6 (1.21) [15]. Even when used with highly numerate individuals, the multiple-choice test format provides good discriminability and remains well balanced [15]. 

In the additional case scenario for the GPs, we presented mammography screening information about disease prevalence and conditional probabilities (sensitivity and 1-specificity) (see Additional file 1). The task was to provide a probability estimate for the positive predictive value. Only the correct answer (9.2%) with an error margin from 8 to 10 was scored as correct; all other responses and missing responses were considered incorrect. Each participant who wrote down a number in this range received one point.

### Outcome measures

 Outcome measures were the total test scores students and GPs achieved on the BNT scenarios, and (for GPs) performance on the hypothetical case scenario. The GPs solved five case scenarios and the students solved four, as mentioned earlier. In addition, baseline characteristics (age, sex, clinical experience for GPs) were obtained from all participants.

### Statistical methods

 We used the standard alpha level of .05 for significance and a power level of .80. Therefore, we needed a sample size of at least 82 participants to detect an effect size showing a minimally important difference (d = .40) [23] in risk literacy levels between clinically experienced GPs and medical students (calculated a priori with G*Power 3.1) [24]. 

Acquired data were entered and analyzed using the statistical software IBM SPSS Statistics 21 (IBM Corp., Armonk, New York, USA). Descriptive means and standard deviations were calculated for participants’ age, and total test scores and frequencies were calculated for sex and for solving the case scenarios. Sample means and frequencies were compared with population means and frequencies using one-sample t-tests and chi-square tests, respectively. We used a Mann–Whitney U test to assess the difference in risk literacy between medical students and GPs as well as the difference between GPs who solved the case scenario successfully and those who did not. In addition, Kendall’s tau correlation coefficient was used to assess the association between BNT scores and successfully solved case scenarios. A chi-square test was used to compare the proportions of GPs and medical students achieving the maximum score. No adjustment for multiple testing was performed.

## Results

### Recruitment process and demographic characteristics

**Fig. 1:**
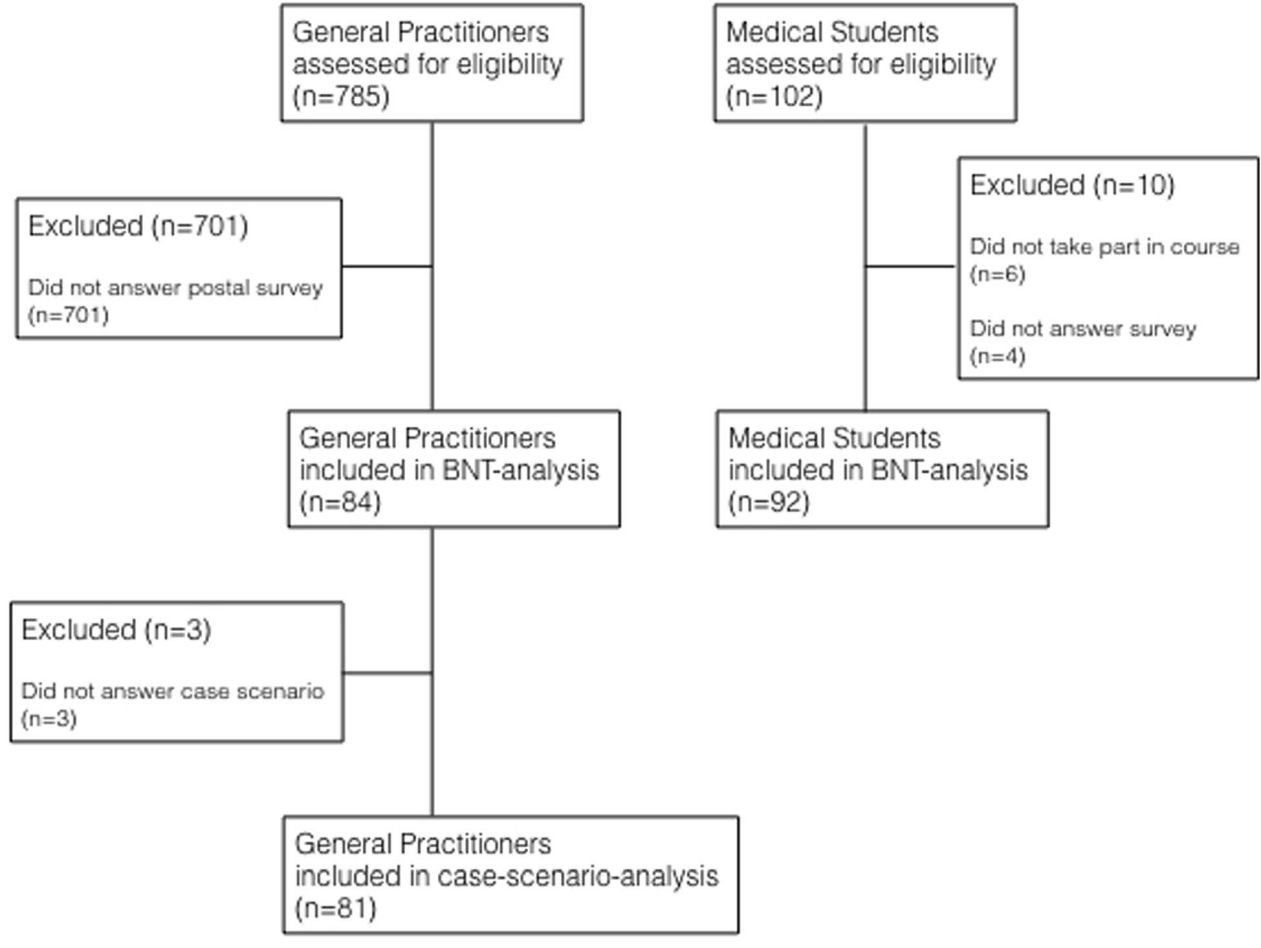
Recruitment process for study samples. BNT, Berlin Numeracy Test

The recruitment process is shown in Fig. 1. We obtained 84 complete data sets (return rate 10.7%) after contacting 785 GPs. The average age of the GPs was 54.7 (SD 8.0) years, and 24 (28.6%) were female. They had an average clinical experience of 25.9 (SD 8.6) years. Ninety-two complete questionnaires from 96 participating medical students were entered into the analysis (return rate 95.8%). The average age of the medical students was 23.2 (SD 4.0) years, and 55 (59.8%) were female. The time needed to complete the test was less than 10 min.

As Table 1 reveals, the baseline characteristics of the study populations and the analyzed samples show no significant differences.

**Table 1:** Baseline characteristics of study populations and analyzed samples

	Study Population	Analyzed Sample	
General Practitioners	Total in the state chamber	Participants	***P***-value
n	4848	84	
Age in years, mean (SD)	55.1 (8.7)	54.7 (8.0)	.65
Female sex, % (n)	33.3 (1613)	28.6 (24)	.36
Clinical experience in years, mean (SD)		25.9 (8.6)	–
**Medical Students**	**Total in the cohort**	**Participants**	***P*-value**
n	102	92	
Age in years, mean (SD)	23.6 (4.5)	23.2 (4.0)	.34
Female sex, % (n)	60.8 (62)	59.8 (55)	.89

*SD* standard deviation

### Primary and secondary outcomes

**Fig. 2:**
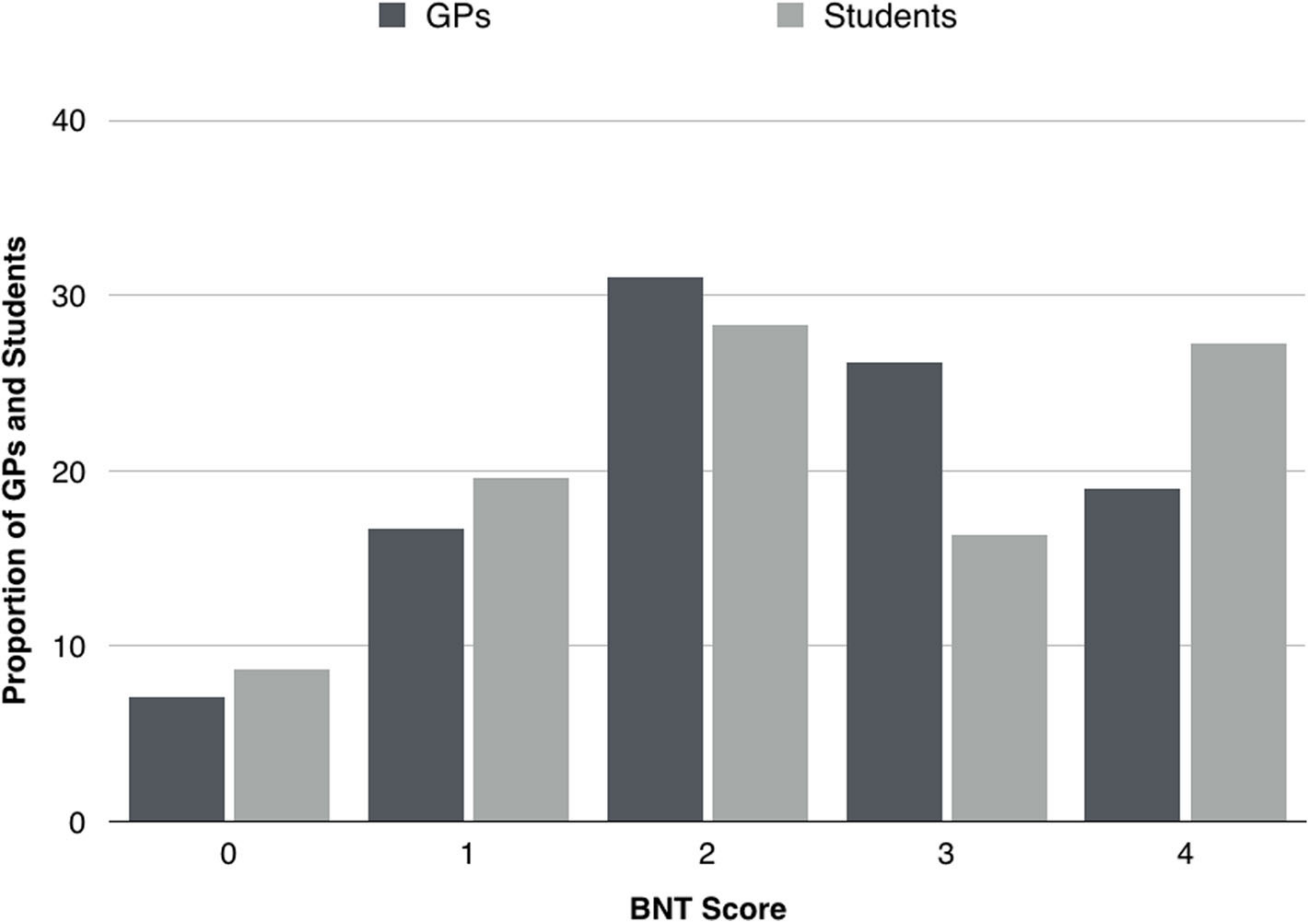
Distribution of Berlin Numeracy Test scores for general practitioners and medical students

After the evaluation of all datasets, three major findings emerged. The first is that participants achieved an average BNT score of 2.34 (SD 1.24) points, which is not meaningfully higher than the average score obtained by Cokely et al. [15] , who reported that a score of 2.2 points was achieved by “highly numerate individuals.” There was only a minimal difference between the scores of GPs (2.33 points [95% confidence intervals [CI] 2.08–2.59]) and medical students (2.34 [95% CI 2.07–2.61]); accordingly, the Mann–Whitney U test results were not significant (P = .983). Figure 2 shows the distribution of scores by group. The proportion of participants achieving the maximum score (4 points) was higher for the student group (27.2% vs. 19.0%, *P* = .20).

 Our second finding was that there were no significant differences between male and female participants (*P* ≥ .05).

Finally, GPs who successfully solved the extra scenario (n = 19, 23.5%) achieved significantly higher scores on the BNT (3.00 points) than did GPs who were unable to solve it (n = 62, 2.11 points,P < .01), a finding reflected in the positive correlation between BNT scores and solving the extra scenario (correlation coefficient Kendall’s tau .31, *P* < .01). Of the GPs, 58 (71.6%) entered values > 80%, a conclusion that greatly overestimates the real predictive value.

## Discussion

### Main findings and implications

Our results show no difference in risk literacy between current students and current GPs. Although showing above-average test results, GPs lack understanding of the underlying success and failure probabilities of mammography screening and therefore cannot accurately relate these to their patients.

The first major finding was that GPs showed above-average test results on the BNT, but did not perform significantly better than medical students. GPs reached a mean risk literacy score of 2.33 points, which according to Cokely et al. [15] is not meaningfully better than the 2.2 points considered average for “highly numerate individuals.” The likelihood that a positive result on a diagnostic test is correct (the test’s positive predictive value) strongly depends on the prevalence of the disease. If prevalence is low, even the best diagnostic test will not be very effective. GPs require accumulated experience in accurate test interpretation and the corresponding risks. However, despite their work in settings with low prevalence for many diseases, GPs show no better risk literacy than do average medical students.

Although the ability to work with numbers is important for physicians, only a handful of studies have explored their numeracy skills. In these studies, physicians have repeatedly shown poor understanding of statistics and quantitative aspects of research [25–28]. They particularly lack the ability to apply Bayesian inferences, which are used to estimate probabilities of a hypothesis in the face of new data [29, 30] [see also Wegwarth & Gigerenzer [31] for a good summary]. In the previously mentioned study by Gigerenzer et al. [16], most gynecologists grossly overestimated the probability of cancer after positive mammography screening results, and their probability estimates were highly variable. Not surprisingly, studies of medical students have shown similar results. In 2002, Sheridan and Pignone provided 62 first-year medical students with three numeracy questions associated with information about the baseline risk for developing a hypothetical disease [32]. They reported that almost one-quarter of the students had trouble performing basic numerical operations. Although showing a relatively high level of numeracy (77% answered all questions correctly), only 61% of students correctly interpreted quantitative data for treatment of a hypothetical disease. The authors concluded that medical students do not understand statistical concepts well, and our findings suggest that this conclusion also applies to fully trained physicians.

We found that GPs performed poorly in applying numeracy skills in the given case scenario. Nearly three-quarters of the GPs entered values of > 80%, a response quite different to the real predictive value. Risk literacy correlated significantly and positively with the ability to solve the case scenario. This is not only a problem with physicians. In the above-mentioned mammography study by Schwartz et al., even the most arithmetically able patients made accurate assessments of risk less than half the time [20]. In reality, physicians and patients are often confronted with additional irrelevant screening evidence, such as improved 5-year survival and increased early detection. Wegwarth et al. found that unfortunately “many physicians did not distinguish between irrelevant and relevant screening evidence”[33], a situation that almost certainly hampers understanding of the benefits and risks of screening. Finally, it can be concluded that GPs lack risk literacy and therefore do not fully understand the numeric estimates of probability associated with routine screening procedures.

In a shared decision-making approach, treatment decisions are made jointly by doctors and patients [34], with the patients’ wishes and needs regarding the therapy receiving considerable weight. This is the core element of patient-centered medicine [34]. Adequate communication of risks of disease and treatment modalities is an important factor in ensuring successful decision-making. Decision-making based on correct understanding of the underlying risks and benefits is central to the physician–patient relationship and is therefore an essential determinant of patient satisfaction and compliance [11, 35]. We were able to show that physicians clearly do not have the necessary numeracy to interpret cancer screening statistics. As Moyer (2012) states, “Expecting them to communicate this information to patients is a stretch.”[36].

Training in risk literacy may help to enhance students’ numeracy skills, but this sometimes does not seem to be the case [37]. GPs who successfully solved the case scenario attained significantly higher BNT scores (3.00 points) than those who did not, a finding reflected in the positive correlation between solving the extra scenario and BNT scores. According to Cohen, a correlation coefficient of >.30 reflects a medium effect size [38]. Therefore, the BNT can help to identify individual weaknesses in numeracy. The BNT is a rapid measure of individual deficiencies in risk literacy and could be used to enhance the effectiveness of training. Several tools exist to increase understanding of risks [39, 40]. However, the introduction of such new training to an already crowded medical studies curriculum remains a challenge.

The most promising way forward is the use of alternative statistical formats, particularly natural frequencies (such as 100 of 1000 instead of 10%) for presenting the risks in screening tests. Therefore, developers of patient decision aids are recommended to preferentially use a natural frequency format for test and screening decisions [41]. The natural frequency method is very effective (Cohen’s d effect size .69; 95% CI 0.45–0.93) and should be used with patients and medical doctors [42]. Authors of publications about screenings should be encouraged by medical journal editors to ensure that the results are presented in terms of natural frequencies to avoid misinterpretation. For all other cases, students should be trained to generate natural frequencies by themselves from provided data.

### Limitations

The main limitation of this study is the difference in questionnaire return rates between GPs and students. Whereas over 90% of the medical students returned questionnaires, far fewer GPs did so. Because the population of interest was a large, geographically dispersed one, a postal questionnaire was our only financially viable option. Although we tried to increase the response rate [43], only about 10% of the contacted GPs replied. Non-response reduces the effective sample size and may have introduced selection bias.

Selection bias severely limits the interpretation of empirical findings. The baseline characteristics provided no indication of systematic differences between the study populations and our analyzed samples. It is likely that if selection bias did occur in this study, it worked *against* the reported findings; that is, it is probable that GPs who answered the postal survey were much more confident about their numeracy skills than those who did not. Our results may therefore actually overestimate GPs’ competence in risk literacy and their understanding of routine screening procedures.

## Conclusions

In this study, we found no difference in risk literacy between current students and current GPs. GPs lack risk literacy and consequently do not fully understand numeric estimates of probability in routine screening procedures.

### Practice points

- The responsibility to help patients understand potential health benefits and risks falls largely to GPs.
- In this study, we found no difference in numeracy skills between current students and current GPs.
- There is a pressing need to help physicians understand and correctly interpret risk information.
- Training on risk literacy during medical school may help, but we also suggest presenting risk information as natural frequencies.

## Supplementary information

**Additional file 1.** Case scenario of mammography screening

## Data Availability

The original data that support the findings of this study are available from https://uni-muenster.sciebo.de/index.php/s/KqWs7nE2rGfnYgr.

## Abbreviations

GP(s): General practitioner(s)
BNT: Berlin Numeracy Test
