# Supplementary material for: Risk literacy assessment of general practitioners and medical students using the Berlin Numeracy Test
Source: BMC Fam Pract. 2020 Jul 14;21:143. doi: 10.1186/s12875-020-01214-w (PMC7362657; doi:10.1186/s12875-020-01214-w)
Supplement: Supplementary file 1 — Additional file 1. Case scenario of mammography screening [file 12875_2020_1214_MOESM1_ESM.pdf]

For early detection of breast cancer, women over 50 years can participate in a mammography screening. About 1% of these women have breast cancer (prevalence). On a mammogram, a breast cancer can be detected with 90% probability (sensitivity). The probability of a false-positive result in a healthy woman is 9%.

What is the probability a woman has breast cancer if she has a positive finding in the mammography?

(Please indicate the likelihood as a percentage; do not use any decimals or commas.)

__________%
